# Post-pandemic respiratory infections in children born during or before the COVID-19 pandemic: a nationwide cohort study in Sweden

**DOI:** 10.1016/j.eclinm.2026.104112

**Published:** 2026-07-28

**Authors:** Henrik Mellström Dahlgren, Huiqi Li, Ailiana Santosa, Leif Dotevall, Karsten Kötz, Fredrik Nyberg

**Affiliations:** a School of Public Health and Community Medicine, Institute of Medicine, Sahlgrenska Academy, University of Gothenburg, Gothenburg, Sweden; b Department of Communicable Disease Control, Region Västra Götaland, Gothenburg, Sweden; c Department of Paediatrics, The Queen Silvia Childrens Hospital, Sahlgrenska University Hospital, Gothenburg, Region Västra Götaland, Sweden; d Department of Paediatrics, Institute of Clinical Sciences, Sahlgrenska Academy, University of Gothenburg, Gothenburg, Sweden

**Keywords:** Respiratory infections, Child health, Immune debt, Non-pharmaceutical interventions, COVID-19

## Abstract

**Background:**

Non-pharmaceutical interventions (NPIs) during the COVID-19 pandemic altered the circulation of respiratory pathogens. Following an initial decline in infections, a resurgence occurred as restrictions eased. We aimed to determine whether reduced early-life exposure to pathogens increased the risk of severe respiratory infections.

**Methods:**

In this longitudinal nationwide population-based study in Sweden, we compared a pandemic birth cohort of children born in Sweden between March 1 and December 31 from 2020 (n = 92,791) with aligned pre-pandemic cohorts born between 1 March and December 31 of 2015 (n = 94,552) and of 2018 (n = 96,074). Children with missing covariate data, death, or emigration before the age of two were excluded (10·5%). Baseline characteristics for the first two years of life were summarized. Outcomes were assessed between ages 2–4 years. Hospitalization for overall and specific lower respiratory tract infections (LRTI) was analysed as a binary outcome using logistic regression.

**Findings:**

Children born in 2020 had markedly lower hospitalisation rates during their first two years of life than both pre-pandemic cohorts. However, between ages 2 and 4, they exhibited significantly higher hospitalisation risk compared with both pre-pandemic cohorts. Adjusted odds ratio (aOR) for hospitalisation in the 2020 versus 2015 cohort were: all-cause LRTI (2·51 (95% CI 2·26–2·79)), influenza (2·99, 2·38–3·79) and respiratory syncytial virus (RSV) (4·36, 3·60–5·31). Similar patterns were observed for all-cause pneumonia and mycoplasma. The 2018 cohort showed a more mixed pattern, with lower risk for pneumonia but higher risk for RSV.

**Interpretation:**

Children born during the COVID-19 pandemic initially had a reduced risk of hospitalisation for respiratory infections, followed by a substantially increased risk in the post-pandemic period compared to children born before the pandemic. These findings suggest that altered early-life microbial exposure due to pandemic-related restrictions may influence subsequent patterns of severe childhood infections through immunological or exposure-related mechanisms and emphasis the need for further research to elucidate underlying mechanisms.

**Funding:**

The SCIFI-PEARLS project has basic fundings from the Swedish government and the county councils, the ALF agreement, the Swedish Research Council for Health, Working Life and Welfare, (Forte), and previously a joint grant from Forte–and the Swedish Research Council for Environment, Agricultural Sciences and Spatial Planning (Formas).

**Research in context**

**Evidence before this study**

We search PubMed up to January 22, 2026, using different combinations of the terms (“respiratory infections” OR “infections” OR “respiratory morbidity”) AND (“immune debt” OR “immune gap” OR “immunity”) AND (“children” OR “birth cohort” OR “paediatric”) AND (“NPI” OR “non-pharmaceutical interventions”) AND (“COVID-19”). No language restrictions were applied. Articles were selected based on title and abstract screening, and reference list of relevant studies were additionally reviewed.

Across the literature, most studies addressed the post-pandemic resurgence of respiratory pathogens through serological assessments, epidemiological descriptions of rebound waves, comparison of pathogen-specific test positivity, or time-series analyses (e.g. immune debt phenomena, disrupted seasonality, delayed epidemics, and increased susceptibility in young children). Although numerous reports of large increases in RSV, *Mycoplasma pneumoniae*, and others respiratory infections following relaxation of NPIs, few studies have examined whether reduced exposure during early childhood leads to increased individual-level risk of severe respiratory morbidity later in childhood. In particular, longitudinal, population-based analyses comparing entire birth cohorts with distinct early-life exposure patterns are largely absent.

Swedish nationwide health and population registers provide a unique opportunity to address this gap.

**Added value of this study**

To our knowledge this is the first nationwide, population-based birth cohort study to examine whether delayed exposure to common respiratory pathogens during the COVID-19 pandemic translates into increased risk of severe respiratory infections requiring hospital admission later in childhood.

In three Swedish birth cohorts, we show that children born during the pandemic experienced substantially fewer hospitalisations for influenza, RSV, pertussis, and *Mycoplasma pneumoniae* during their first two year of life, consistent with reduced circulation of respiratory pathogens during the NPI period. However, between ages two and four, these children exhibited markedly higher risks of hospitalizations due to respiratory infections compared with pre-pandemic cohorts. The findings confirm that pandemic related restrictions significantly influenced the spread of viral and bacterial pathogens, even though Sweden never implemented a total lockdown, and preschools remained open throughout the pandemic. Our results suggest that delayed exposure to infectious agents may cause a rebound and increase the risk of severe infections in early childhood.

**Implications of all the available evidence**

The post-pandemic surge of hospitalizations due to various respiratory infections highlights the importance of broad, real-time surveillance for respiratory pathogens to anticipate and manage rebound waves. Our results highlight the long-term consequences NPIs may have on immunity development and infection risk in young children.

These findings underscore the need for preparedness strategies to mitigate similar effects in future pandemics. The results emphasize the need to quickly get treatments and vaccines in place for an epidemic so that NPIs can be relaxed to minimize unintended consequences and they highlight the need for further research to elucidate underlying mechanisms.

## Introduction

In the aftermath of the COVID-19 pandemic many countries in Europe and United States reported an increase of respiratory infections, especially among children under 10 years of age.^1^, ^2^, ^3^ The sharp increase of paediatric infectious diseases also revealed more unusual and severe clinical manifestations such as parapneumonic effusions.^2^^,^^4^^,^^5^ In December 2022, the World Health Organisation advised European countries to be vigilant for invasive group A Streptococcus infections (iGAS) among children, to diagnose and treat patients promptly and raise awareness among the clinicians and the public.^6^ These reports raised concerns not only about increased transmission but also whether post-pandemic infections in children may cause more severe disease manifestations and an increased need for hospital-based care.

The cause of resurgent respiratory infections has been assumed to be multifactorial. When the COVID-19 pandemic emerged, Non-pharmaceutical interventions (NPI) such as social distancing, quarantine and other containment measures were implemented in order to mitigate the strain on the healthcare system and society. One of the consequences of the combined measures in Sweden as elsewhere was a substantial decline of other infectious diseases during the pandemic period, with a later sharp surge of infectious diseases in the post-pandemic period.^7^^,^^8^ This has often been explained by the concept of “immune debt”, referring to reduced population-level immunity resulting from decreased exposure to common pathogens during prolonged periods of infection control measures.^9^ However, empirical individual-level evidence linking delayed early-life exposure to later severe infection risk is limited. Although, other factors contributing to more severe bacterial infections have also been suggested, such as the emergence of more virulent strains of e.g. *Streptococcus pyogenes* which are likely to have driven of the observed increase of iGAS, and the overall rise in viral respiratory tract infections after the pandemic, as those are known to precede bacterial lower respiratory tract infections (LRTI).^10^ Altogether, the disruption of usual paediatric infections pattern has mainly been understood from a trinity of altered pathogenesis, immune debt and viral-bacterial synergy.

The Swedish mitigation policy was unique and came to be widely debated since no law-enforced general lockdown or mandatory mask-wearing was implemented. Containment relied largely on strong social distancing and some mask recommendations and some legal restrictions regarding public gatherings.^11^^,^^12^ Early childhood education (age 1–5 years), commonly referred to as preschool, remained open throughout the pandemic in Sweden, and therefore this period and context are interesting to evaluate. During a pandemic there is a need to suppress the spread of infectious agents and yet balance this with other potential negative consequences for individuals and society. One potential drawback is reduced herd immunity with later increase of other infections and potentially increased severity of such infections.

Despite extensive descriptive surveillance of post-pandemic infections surges, few studies have examined whether delayed exposure to common infectious agents during early childhood translates into an increased individual-level risk of severe respiratory infections later in childhood. In particular, evidence from longitudinal, population-based studies comparing birth cohorts with differing early-life exposure patters remains scare. Swedish nationwide health and population registers provide a unique opportunity to address this gap. Therefore, using nationwide individual-level high quality register data on three different birth cohorts in the Swedish population, this study aimed to assess whether delayed exposure to infectious agents during early childhood are associated with increased risk of severe respiratory infections. We hypothesized that children born during the pandemic may have had a higher risk of hospital admissions due to respiratory infections compared to pre-pandemic cohorts, possibly reflecting delayed immune priming in early life and altered exposures for respiratory pathogens.

## Methods

### Data sources

This analysis is part of the SCFI-PEARL (Swedish COVID-19 Investigation for Future Insights—a Population Epidemiology Approach using Register Linkage) project.^13^ The data cover the entire Swedish population since 2015. SCIFI-PEARL links data from several high-quality health and administrative registers on a regularly updated basis using the unique Swedish personal identity number.^13^^,^^14^ The data are pseudonymized prior to delivery to the investigators. Sociodemographic variables came from the Total Population Register (TPR),^15^ the Longitudinal Integrated Database for Health Insurance and Labor Market Studies (LISA)^16^ and the Dwelling Units Register at Statistics Sweden. Area-level socioeconomic data were also collected from Statistics Sweden open database and measure the status by each birth year. Variables related to LRTI and other respiratory infections and prior comorbidities were obtained from the National Patient Register (NPR).^17^ The NPR has nationwide coverage and includes all inpatient care and outpatient specialist care visits. Information on maternal age, gestational age, birth weight and maternal smoking were retrieved from the Medical Birth Register.^18^ Time-series data on notifications of respiratory syncytial virus infection (RSV) and influenza were retrieved from the open database of the Public Health Agency,^19^ representing the weekly number of reported cases across all age groups and were used descriptively to contextualize epidemic timing.

### Study design and study population

This was a nationwide-population-based register cohort study comparing three birth cohorts. The study population included all children born in Sweden in 2015, 2018, and 2020, respectively. To align with the pandemic restriction period and to avoid systematic misclassification of the exposure window, as children born in January and February would have spent a disproportionate share of their first two months of life before the period of interest, we included only children born between March 1 and December 31 in each of these years. A total of 33,211 children were excluded due to missing covariate data, death, or emigration before the age of two (Supplementary Figure S1), leaving a final study population of 283,417 children. To assess whether data were missing at random, we performed a separate analysis of excluded individuals (see Supplementary Tables S2–S4).

### Exposure

**Fig. 1:**
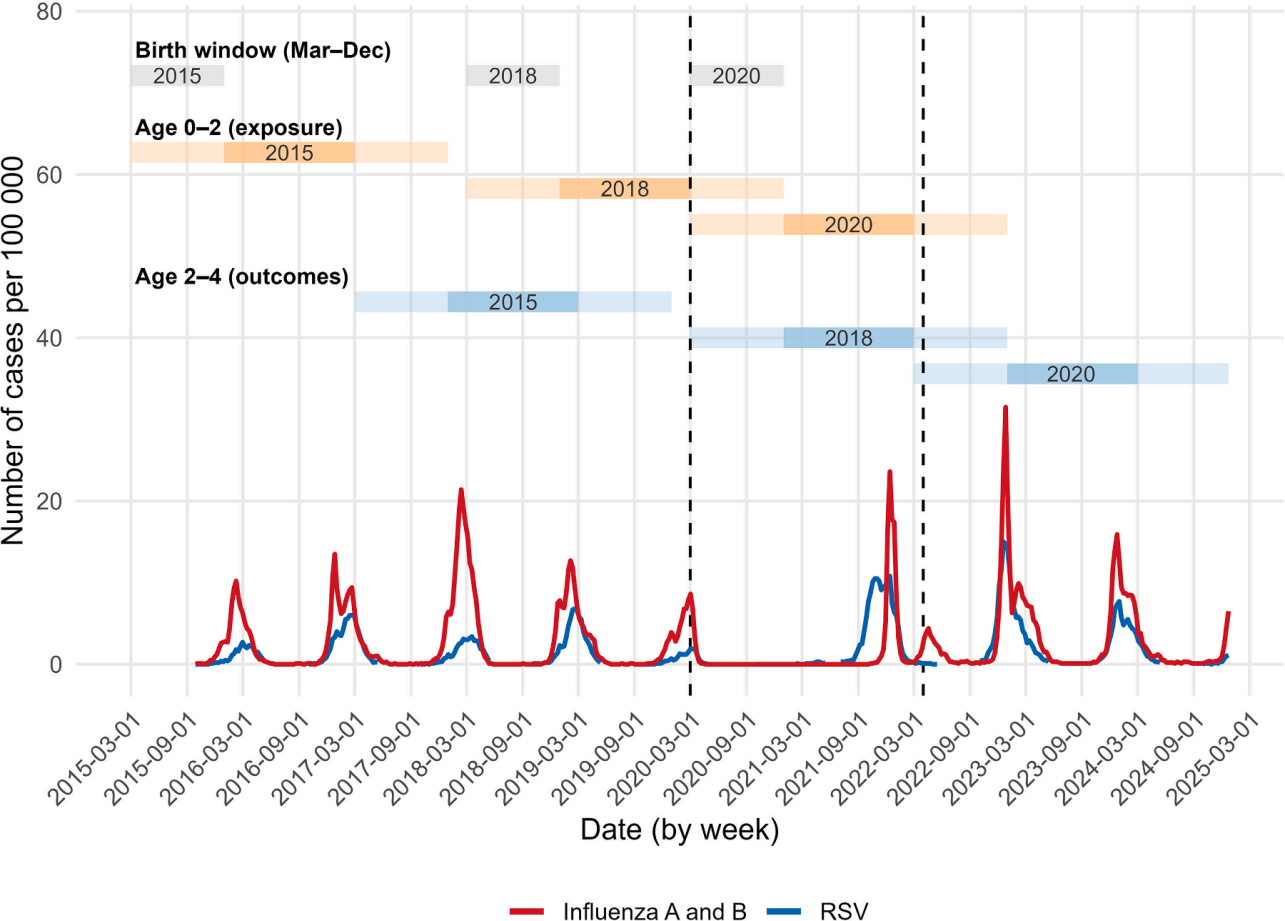
Timeline of birth cohorts and exposure and outcome windows for children born in Sweden between 1 March and 31 December in 2015, 2018 and 2020 in relation to weekly incidence of respiratory syncytial virus (RSV; blue) and influenza A and B (red) per 100,000 of the total population (all ages), 1 March 2015–31 December 2024. Bars indicate birth and exposure/outcome windows; darker segments denote periods during which all children in a cohort were concurrently within the window. Dashed vertical lines mark the period of COVID-19 restrictions (2020-03-01 to 2022-04-01).

The study population formed three cohorts based on birth year, reflecting different exposure to the pandemic-related control measures during the early years. The 2015 cohort constitutes the pre-COVID-19 unexposed group. The 2018 cohort represents an intermediate group, born before the pandemic but aged 2–4 years during the restriction period. The 2020 cohort represents the COVID-19 group, born during the restrictions and 2–4 years old during the post-pandemic period. Fig. 1 illustrates the timeline for the three birth cohorts, along with the exposure and outcome windows, in relation with COVID-19 restrictions, seasonal influenza and RSV epidemics from March 2015 through December 2024.

### Follow-up and outcomes

The follow-up started from the day when each child reached age 2 (baseline) to the day they turned age 4, death, emigration or end of follow-up, whichever occurred first. This age window represents a period when most Swedish children attend preschool, a period of high social interaction and susceptibility to respiratory infections. The outcomes were hospitalisations for all-cause LRTI, hospitalisations for specific lower manifestations of respiratory infections (all-cause pneumonia, bacterial pneumonia and pyothorax) and hospitalisations for pathogen specific infections (Invasive pneumococcal infection, influenza, RSV, mycoplasma and pertussis). Outcomes were identified using ICD-10 codes in NPR for hospital admission (Supplementary Table S1).

### Other covariates

Gestational age, maternal smoking and sex were recorded at birth and household size, place of residence and maternal education were assessed at end of calendar year before birth. Gestational age was categorized according to week + days as.

• Normal (37 + 0–41 + 6),
• Extremely preterm (22 + 0–27 + 6),
• Very preterm (28 + 0–31 + 6),
• Moderately preterm (32 + 0–36 + 6),
• Post-term (≥42 + 0).

Maternal country of birth was categorized in into three groups according to the World Bank classification^20^; born in Sweden, born in middle–high income countries (MHICs) and born in low-middle income countries (LMICs) (Supplementary Table S2). Maternal education was defined as the highest level attained, classified as primary (compulsory and lower secondary), secondary (upper secondary) or tertiary (higher education). Household size was based on number of registered persons in the mother's household (including the child) and categorized as ≤3 persons, 4–7 persons and 8+ persons.

We used the regional statistics areas (RegSO) which represents a aggregation of small geographic area units, i.e. the neighbourhood, as a proxy for the mothers’ socioeconomic positions according to her place of residence, and we used the Area Type classification which is a composite measurement based on three indicators: proportion of individuals with low economic standards, proportion with tertiary education and proportion receiving financial assistance for ≥10 months and/or unemployed >6 months.^21^

Comorbidities assessed at baseline (from birth to age two) were obtained from NPR and included asthma (ICD-10 code J45), cancer (ICD-10 codes C00–C97), cerebral palsy (ICD-10 code G80) and bronchopulmonary dysplasia (ICD-10 code P27·1). Congenital malformations were defined as abnormalities recorded under ICD-10 codes Q00–Q99, with exception for minor anomalies and unspecified codes that should not reported to the Swedish registry for monitoring congenital malformations and chromosomal abnormalities.^22^

### Statistical analysis

Baseline characteristics were summarized using frequencies and percentages or median (inter quartile range). Initial comparisons of outcomes across the three cohorts were performed using chi-squared tests, comparing all three birth cohorts simultaneously in a single global test. Hospitalisation due any-cause LRTI, pneumonia, influenza, mycoplasma and RSV were further analysed using logistic regression models, except for pertussis, invasive pneumococcal infections and pyothorax due to small numbers. An unadjusted model and a model adjusted for sociodemographic factors and comorbidities were used. In this analysis 47 individuals with missing data concerning maternal birth country were additionally excluded. Results are presented as odds ratios (OR) with 95% confidence interval (CIs).

To account for potential confounding by periods of high infection rates rather than delayed exposure, a sensitivity analysis was conducted in a subset of children from small households (≤3 persons at birth), where infection transmission would be assumed to be less likely.

Following peer review, we conducted two post-hoc analyses. First, cumulative incidence over the whole timespan of the three cohorts was examined, as described above. Second, a segmented regression of the long-term trend and the change in trend during the pandemic years was conducted by using open data for hospitalisations rates for pneumonia and influenza among children aged 0–4 from the National Board of Health and Welfare.

All statistical analyses were conducted using R version 4·3·3 (The R Foundation for statistical Computing, Vienna Austria).

### Ethics

The study has ethical approval from the Swedish Ethics Review Authority, decision no. 2020-01800 with subsequent amendments. Informed consent was waived as the study was based on pseudonymized register data.

### Role of the funding source

The funders had no involvement in study design, data collection, data analyses, data interpretation, or the writing of the report.

## Results

A total of 283,417 children were included: 94,552 (33%) in 2015 birth cohort, 96,074 (34%) in the 2018 cohort and 92,791 (33%) in the 2020 cohort. Most birth and maternal characteristics were comparable between the groups, although the 2020 cohort had fewer post-term births (3·2% versus 7·3% in 2015) and a lower prevalence of maternal smoking (5·2% versus 8·9% in 2015) (Table 1).

**Table 1:** Distribution of birth, maternal and sociodemographic characteristics among children born in Sweden between 1 March and 31 December in 2015, 2018 and 2020.

Characteristic	Birth cohort		
	2015 N = 94,552^a^	2018 N = 96,074^a^	2020 N = 92,791^a^
Sex			
Male	49,005 (52%)	49,350 (51%)	47,730 (51%)
Female	45,547 (48%)	46,724 (49%)	45,061 (49%)
Birth weight in grams	3535 (3190, 3880)	3525 (3185, 3865)	3530 (3190, 3860)
Gestation age in days	280 (273, 287)	280 (273, 286)	280 (273, 286)
Gestation age (weeks + days)			
Normal (37 + 0 – 41 + 6)	82,504 (87%)	84,661 (88%)	84,999 (92%)
Extremely preterm (22 + 0 – 27 + 6)	232 (0·2%)	202 (0·2%)	218 (0·2%)
Very preterm (28 + 0 – 31 + 6)	488 (0·5%)	464 (0·5%)	464 (0·5%)
Moderately preterm (32 + 0 – 36 + 6)	4402 (4·7%)	4341 (4·5%)	4097 (4·4%)
Post-term (≥42 + 0)	6926 (7·3%)	6406 (6·7%)	3013 (3·2%)
Residential area type			
5. Very good socioeconomic conditions	9990 (11%)	9435 (9·8%)	8263 (8·9%)
4. Good socioeconomic conditions	48,576 (51%)	50,450 (53%)	49,963 (54%)
3. Mixed areas	20,625 (22%)	20,774 (22%)	20,310 (22%)
2. Socioeconomic challenges	7613 (8·1%)	8272 (8·6%)	7765 (8·4%)
1. Major socioeconomic challenges	7748 (8·2%)	7143 (7·4%)	6490 (7·0%)
Household size (numbers of persons)			
≤3	35,908 (38%)	36,901 (38%)	36,413 (39%)
4–7	55,540 (59%)	56,023 (58%)	53,797 (58%)
≥8	3104 (3·3%)	3150 (3·3%)	2581 (2·8%)
Maternal birth country			
Born in Sweden	68,839 (73%)	67,300 (70%)	65,149 (70%)
Born in MHIC	15,096 (16%)	14,839 (15%)	14,042 (15%)
Born in LMIC	10,605 (11%)	13,917 (14%)	13,583 (15%)
Unknown	12 (<0·1%)	18 (<0·1%)	17 (<0·1%)
Maternal age, years Median (Q1, Q3)	30·0 (27·0, 34·0)	30·0 (27·0, 34·0)	31·0 (28·0, 34·0)
Maternal smoking			
Nonsmoker	84,830 (90%)	87,666 (91%)	85,400 (92%)
Smoking according to pregnancy	8397 (8·9%)	5960 (6·2%)	4855 (5·2%)
Missing	1325 (1·4%)	2448 (2·5%)	2536 (2·7%)
Maternal education			
Tertiary	49,168 (52%)	51,046 (53%)	51,282 (55%)
Secondary	33,159 (35%)	32,769 (34%)	30,629 (33%)
Primary	12,225 (13%)	12,259 (13%)	10,880 (12%)

Q1, first quartile; Q3, third quartile 3; MHIC, middle–high income countries; LMIC, low-middle income countries.

a n (%); Median (Q1, Q3).

Baseline prevalence of risk factors, i.e. conditions registered up till the second birthday, was similar across the cohorts with the exception of Congenital risk factors and asthma, were the latter had a prevalence of 4·4% in the 2020 cohort, compared with 5·5% in the 2018 cohort and 6·8% in the 2015 cohort (Table 2). At baseline, the 2020 cohort had substantial lower rates of hospitalisation due to all-cause LRTI, all-cause pneumonia, influenza, RSV, mycoplasma and pertussis. Admissions rates for bacterial pneumonia were similar across cohorts. Hospitalisation due to invasive pneumococcal infection and pyothorax was rare in all groups, although slightly higher in the 2020 cohort (Table 2).

**Table 2:** Baseline characteristics, medical risk factors, and cumulative incidence of respiratory infection related hospitalisations at age 2 years among children born in Sweden between 1 March and 31 December in 2015, 2018 and 2020.

	Birth cohort			p-value^b^
	2015 N = 94,552^a^	2018 N = 96,074^a^	2020 N = 92,791^a^	
Clinical risk factors at baseline				
Congenital risk factors	5625 (5·9%)	6137 (6·4%)	6212 (6·7%)	<0·001
Bronchopulmonary dysplasia	268 (0·3%)	189 (0·2%)	208 (0·2%)	<0·001
Asthma	6428 (6·8%)	5300 (5·5%)	4051 (4·4%)	<0·001
Cerebral palsy	96 (0·1%)	99 (0·1%)	87 (<0·1%)	0·8
All forms of cancer	47 (<0·1%)	34 (<0·1%)	54 (<0·1%)	0·7
Hospitalisations due to respiratory infections				
All-cause Lower respiratory tract infection	2210 (2·3%)	2653 (2·8%)	1167 (1·3%)	<0·001
All-cause Pneumonia	724 (0·8%)	602 (0·6%)	555 (0·6%)	<0·001
Pneumonia–bacterial	219 (0·2%)	206 (0·2%)	188 (0·2%)	0·4
Invasive pneumococcal infection	20 (<0·1%)	25 (<0·1%)	30 (<0·1%)	0·3
Pyothorax	2 (<0·1%)	7 (<0·1%)	14 (<0·1%)	0·007
Influenza	147 (0·2%)	208 (0·2%)	39 (<0·1%)	<0·001
Mycoplasma	99 (0·1%)	127 (0·2%)	62 (<0·1%)	<0·001
Pertussis	33 (<0·1%)	30 (<0·1%)	2 (<0·1%)	<0·001
RSV	954 (1·0%)	1521 (1·6%)	497 (0·5%)	<0·001

RSV, respiratory syncytial virus.

a n (%).

b Pearson's Chi-squared test.

Overall, the 2020 cohort exhibited significantly higher admissions rates during follow-up at ages 2–4 compared to both pre-pandemic cohorts (Table 3). For example, hospitalisation due to all-cause LRTI occurred in 1·3% of the children in the 2020 cohort, compared to 0·6% and 0·5% in the 2015 and 2018 cohort respectively. Similarly, hospitalization due to all-cause pneumonia, bacterial pneumonia and mycoplasma infection were more frequent in the 2020 cohort than in the 2015 and 2018 cohorts. Although invasive pneumococcal infections were infrequent in all groups, the incidence was modestly elevated in the 2020 cohort. Few pyothorax admissions were observed but showed a clear increase in the 2020 cohort as 29 individuals were registered (corresponding to a cumulative incidence of 31·3 per 100,000), compared to 6 (6·3 per 100,000) and 9 (9·3 per 100,000) in the 2015 and 2018 cohort respectively. The viral infections, influenza and RSV also demonstrated a pronounced difference with higher rates of hospitalisations in the 2020 cohort. Admissions due to pertussis were essentially absent. All-cause death remained low and comparable across the cohorts (Table 3).

**Table 3:** Cumulative incidence of infection-related hospitalisations and all-cause death between ages 2 and 4 years across birth cohorts, among children born in Sweden between 1 March and 31 December in 2015, 2018 and 2020.^c^

Respiratory outcome	Birth cohort			p-value^b^
	2015 N = 94,552^a^	2018 N = 96,074^a^	2020 N = 92,791^a^	
All-cause Lower respiratory tract infection	527 (0·6%)	528 (0·5%)	1228 (1·3%)	<0·001
All-cause Pneumonia	413 (0·4%)	321 (0·3%)	835 (0·9%)	<0·001
Pneumonia–bacterial	147 (0·2%)	104 (0·1%)	331 (0·4%)	<0·001
Invasive pneumococcal infection	12 (<0·1%)	17 (<0·1%)	35 (<0·1%)	<0·001
Pyothorax	6 (<0·1%)	9 (<0·1%)	29 (<0·1%)	<0·001
Mycoplasma	25 (<0·1%)	39 (<0·1%)	91 (<0·1%)	<0·001
Influenza	98 (0·1%)	38 (<0·1%)	278 (0·3%)	<0·001
RSV	130 (0·1%)	246 (0·3%)	513 (0·6%)	<0·001
Pertussis	0 (0%)	0 (0%)	2 (<0·1%)	
All-cause death	17 (<0·1%)	19 (<0·1%)	17 (<0·1%)	>0·9

RSV, respiratory syncytial virus.

a n (%).

b Pearson's Chi-squared test.

c Specifically born between March and December.

The two comparisons cohorts, the 2015 and 2018 cohorts, were largely similar in hospitalisation rates for respiratory infections, with the exception of influenza and RSV: the 2018 cohort had 38 hospitalisations for influenza (corresponding to a cumulative incidence of 39·6 per 100,000) compared to 98 (103·6 per 100,000) in the 2015 cohort, while RSV hospitalisations were more frequent in the 2018 cohort 246 (256 per 100,000) compared with 130 (137·5 per 100,000) in the 2015 cohort (Table 3).

In logistic regression models, crude and adjusted odds ratios (OR) were similar across all outcomes, indicating that sociodemographic and clinical factors had only a minor confounding effect. For all-cause LRTI, no difference was observed between the 2015 and 2018 cohorts in either crude or adjusted models. In contrast, the 2020 cohort had substantially higher risks, with an adjusted OR (aOR) of 2·51 (95% CI 2·26–2·79) for LRTI-hospitalisation compared to the 2015 cohort. This pattern was consistent across all specific outcomes as the 2020 cohort exhibited significantly increased risks of hospitalisation also after adjusting for confounding factors. For the 2018 cohort, the pattern was more mixed, with lower risk than the 2015 cohort for all-cause pneumonia, bacterial pneumonia and higher for RSV (aOR 1·96, 95% CI 1·59–2·44) (Table 4).

**Table 4:** Logistic regression models for hospitalisation due to different respiratory tract infections among children born in Sweden between 1 March and 31 December in 2015, 2018 and 2020, crude and adjusted for sociodemographic and for clinical risk factors (sex, asthma, cancer, congenital malformations, bronchopulmonary dysplasia, cerebral palsy, gestational age, maternal smoking, maternal education, maternal birth country, residential area type, household size), with 2015 as the reference category and all three cohorts in the model.

Respiratory outcome	Crude OR^a^ (95% CI^a^)	Adjusted OR (95% CI)
All-cause lower respiratory tract infection		
2015 (ref)	1·00	1·00
2018	0·99 (0·87–1·11)	1·01 (0·90–1·14)
2020	2·39 (2·16–2·65)	2·51 (2·26–2·79)
All-cause pneumonia		
2015 (ref)	1·00	1·00
2018	0·76 (0·66–0·88)	0·78 (0·67–0·90)
2020	2·07 (1·84–2·33)	2·16 (1·92–2·44)
Pneumonia–bacterial		
2015 (ref)	1·00	1·00
2018	0·70 (0·54–0·89)	0·70 (0·54–0·90)
2020	2·30 (1·90–2·80)	2·34 (1·93–2·86)
Influenza		
2015 (ref)	1·00	1·00
2018	0·38 (0·26–0·55)	0·38 (0·26–0·56)
2020	2·90 (2·31–3·66)	2·99 (2·38–3·79)
Mycoplasma		
2015 (ref)	1·00	1·00
2018	1·54 (0·94–2·57)	1·57 (0·96–2·64)
2020	3·71 (2·42–5·90)	3·89 (2·53–6·21)
RSV		
2015 (ref)	1·00	1·00
2018	1·86 (1·51–2·31)	1·96 (1·59–2·44)
2020	4·04 (3·34–4·91)	4·36 (3·60–5·31)

RSV, respiratory syncytial virus.

a OR = Odds Ratio, CI = Confidence Interval.

Individuals excluded due to missing data were more likely to have mothers with a foreign background, and a larger proportion of exclusions occurred in the 2015 cohort. However, the distribution of baseline characteristics and the pattern of outcomes were consistent across birth cohorts (Supplementary Tables S2–S4). In the sensitivity analyses restricted to children from small households (≤3 persons at birth), the same pattern emerged, showing a higher hospitalisation rate for all-cause LRTI, all-cause pneumonia, bacterial pneumonia, mycoplasma infections, influenza and RSV in the 2020 cohort compared to the 2015 and 2018 cohorts (Supplementary Table S5).

When viewed across the entire first four years of life, the 2020 birth cohort showed markedly higher cumulative incidence of bacterial pneumonia, invasive pneumococcal infection, and pyothorax compared with the 2015 and 2018 cohorts, while RSV hospitalisations were notably lower (Table 5). All-cause pneumonia was also more frequent in the 2020 cohort, whereas pertussis was substantially lower.

**Table 5:** Cumulative incidence of infection-related hospitalisations between ages 0 and 4 years across birth cohorts, among children born in Sweden between 1 March and 31 December in 2015, 2018 and 2020.^c^

Respiratory outcome	Birth cohort			p-value^b^
	2015 N = 94,552^a^	2018 N = 96,074^a^	2020 N = 92,791^a^	
All-cause Lower respiratory tract infection	2620 (2·8%)	3105 (3·2%)	2289 (2·5%)	<0·001
All-cause Pneumonia	1097 (1·2%)	900 (0·9%)	1346 (1·5%)	<0·001
Pneumonia–bacterial	356 (0·4%)	304 (0·3%)	511 (0·6%)	<0·001
Invasive pneumococcal infection	31 (<0·1%)	42 (<0·1%)	64 (<0·1%)	<0·001
Pyothorax	8 (<0·1%)	16 (<0·1%)	40 (<0·1%)	<0·001
Mycoplasma	123 (<0·1%)	166 (<0·2%)	153 (<0·1%)	0·043
Influenza	243 (0·3%)	245 (<0·3%)	316 (0·3%)	<0·001
RSV	1072 (1·1%)	1752 (1·8%)	993 (1·1%)	<0·001
Pertussis	33 (<0·1%)	30 (<0·1%)	4 (<0·1%)	<0·001

RSV, respiratory syncytial virus.

a n (%).

b Pearson's Chi-squared test.

c Specifically born between March and December.

The segmented regression based on open aggregated annual data identified a breakpoint at year 2020, with a clear decreasing trend prior to the pandemic, followed by a more volatile phase thereafter (Supplementary Figure S2).

## Discussion

In this comprehensive nationwide study in Sweden encompassing three distinct birth cohorts, we observed that children born during the COVID-19 pandemic (2020 cohort) initially experienced markedly fewer hospital admissions for influenza, RSV, pertussis and mycoplasma infections during their first two years compared with pre-pandemic cohorts. However, between ages two and four, these children exhibited substantially higher risks of hospitalisations due to respiratory infections. The cumulative incidence over the first four years of life shows a mixed pattern, partly consistent with a delay in respiratory infection rather than a net increase per se. Total LRTI incidence was lower in the 2020 cohort; however, COVID-19 is not captured within this diagnostic category, as the definition was established for comparability with pre-pandemic cohorts, and it is plausible that a proportion of lower respiratory tract episodes were attributed to COVID-19 and therefore not reflected in these figures. RSV hospitalisation rates returned to pre-pandemic levels, consistent with a redistribution of infections across time rather than an increase in burden. However, the cumulative incidence of more severe bacterial complications, including pyothorax and invasive pneumococcal infections, was substantially higher in the 2020 cohort, suggesting that children born during the pandemic may have experienced qualitatively more sever infectious episodes. Whether this reflects altered immune priming, changes in pathogen virulence or residual confounding by healthcare seeking behaviour warrants further investigation. These findings directly support our hypothesis of increased post-pandemic respiratory hospitalisation risk among children born during the pandemic. The findings confirm that pandemic-related restrictions significantly influenced the spread of viral and bacterial pathogens, even though Sweden never implemented a total lockdown, and preschools remained open throughout the pandemic. Our results suggest that such delayed exposure to infectious agents can cause a rebound and increase the risk of severe infections in early childhood.

The first four years of life represent a critical window for primary infections and immune system maturation as children transition from toddlerhood to preschool age.^23^ This may partly explain the observed surge in hospitalisations. These observations align with previous research indicating that Non-pharmaceutical interventions (NPIs) during the COVID-19 pandemic created an “immunity gap” with a decline of antibodies for several infectious pathogens, especially in children aged 3–4 years.^24^^,^^25^ The circulations of viruses and bacteria were low for an extended period because of NPIs, which led to reduced pathogen-specific adaptive immunity within the population.^26^ Consequently, young children remained immunological naïve due to the delayed primary infections, while slightly older preschool children became susceptible as immunity waned in the absence of recent re-infection.^27^ Importantly, the resurgence of infections in Sweden, despite less stringent NPIs than in many other countries, suggest that this phenomenon is not solely dependent on the stringency of restrictions. However, while immune debt represents a plausible explanation for the observed increase in hospitalisations, we cannot exclude the possibility that some of the observed increase reflects changes in exposure besides susceptibility.

Although the cohorts were broadly comparable, some differences were noted. The 2020 pandemic cohort included fewer post-term births, likely reflecting new induction protocols introduced at 41 weeks in the 2020s.^28^ Maternal smoking prevalence was lower in later birth cohorts, consistent with a shift among nicotine users toward increase use of Swedish snuff among pregnant women.^29^ Furthermore, asthma prevalence at the 2-year baseline was lower in the 2018 and 2020 cohorts compared with 2015. A previous study has shown that asthma incidence among children in the Nordic countries may have plateaued,^30^ though this warrants further investigation.

Interestingly, hospitalisations for bacterial pneumonia during the first two years were similar across cohorts, consistent with a report of unchanged pneumococcal carriage rate during the pandemic.^31^ This supports the hypothesis of an important viral-bacterial interaction, as the subsequent resurgence of bacterial infections coincided with increased viral activity.^10^^,^^32^, ^33^, ^34^ Between ages two and four, the 2020 pandemic cohort demonstrated significantly higher incidence of hospitalisation for all investigated outcomes except pertussis, which is targeted by vaccination programs. RSV and influenza exhibited the largest post-pandemic resurgences, and these viruses likely act as drivers for secondary bacterial infections. This underscores the importance of targeted interventions, such as vaccination and prophylaxis, for children at higher risk of severe infections.

The observed crude differences between the cohorts were confirmed in the regression analyses adjusting for potential confounders. Notably, some differences between the two pre-pandemic cohorts were also seen: the 2018 cohort had lower rates of influenza and pneumonia, but higher RSV rates compared to the 2015 cohort. This could indicate that influenza may play an important role in pneumonia in this age group, which necessitates further studies.

Whether the observed excess in hospitalisations is driven by immunity or by heightened post-pandemic exposure is difficult to disentangle given their intertwined causal relationship at the population level. In Sweden, children are entitled to preschool from one years of age, yet approximately 50% are enrolled in their first year,^35^ meaning a substantial proportion of the youngest children have limited exposure to circulating pathogens outside the home. Importantly, restricting the analyses to children in smaller households without older siblings and/or other potential in–home vectors yielded similar results. Smaller households plausibly entail reduced opportunities for pathogen exposure, supporting the interpretation that differential immunity, rather than exposure alone, accounts for the observed pattern.

A major strength of this study is the use of high-quality registers covering the entire Swedish population. However, certain limitations should also be acknowledged. Our analyses estimate differences in observed hospitalisation risk but do not directly model transmission dynamics, pathogen exposure, or immunological mechanisms; accordingly, the development of mechanistic models represents an important direction for future research. Restricting the cohort to children born between March and December avoids exposure misclassification but may introduce a degree of seasonal bias, as births in January and February are not represented, and adjustment for seasonality was not considered appropriate. As the cohorts were predefined to capture differences in early-life exposure with a focus on understanding the fate of the 2020 birth cohort, we did not analyse overall temporal trends or whether the pandemic caused a temporary disruption or marked a long-term shift in trends for respiratory infections in children, an issue that warrants further investigation. However, the complementary post-hoc analysis based on open aggregated annual data indicated a clear decreasing trend prior to the pandemic, followed by a more volatile phase thereafter, an overall pattern that is fairly well known already from across the world. Nevertheless, a more robust analysis would require additional yearly time points to reliably estimate changes in trend from 2020 onwards. Missing data on gestational age and birth weight led to the exclusion of approximately 10 percent of the population, which will affect the absolute numbers, although the large sample size supports generalisability. The outcome patterns in the analysis of excluded individuals mirrored those observed in the main analysis, suggesting that the missingness was approximately random, and we do not expect this to have affected our findings. Outcome misclassification is possible when relying on ICD-10 codes, particularly for specific manifestations such as pyothorax and etiological outcomes such as invasive pneumococcal infections. The absence of laboratory data limited pathogen-specific analyses and interpretation. Vaccination data were also unavailable, although coverage for pertussis and pneumococcal infections in Sweden is generally high as almost 95% of children complete the vaccinations program.^36^ The covariates ethnicity and race are not recorded in Swedish national registers. As a proxy we included maternal country of birth and a socioeconomic classification of the residential place, which partly captures socioeconomic and demographic variation related to migration background. Residual confounding cannot be excluded, particularly due to potential changes in healthcare-seeking behaviour and hospital admission practices in the post-pandemic period. Some caution is warranted when generalizing our findings beyond Sweden, especially given the less restrictive pandemic measures.

In summary, the COVID-19 pandemic provided a unique opportunity to study the effects of altered microbial exposures during early childhood. The post-pandemic surge of hospitalisations due to various respiratory infections highlights the importance of broad, real-time surveillance for respiratory pathogens to anticipate and manage rebound waves. Our findings offer important insights into the long-term consequences of NPIs on immunity and infection risk, emphasizing the need for preparedness strategies to mitigate similar effects in future pandemics. Future studies with longer follow-up and access to microbiological and immunological data are needed to further elucidate underlying mechanisms.

Children born during the COVID-19 pandemic initially had a reduced risk of hospitalisation for respiratory infections, followed by a markedly increased risk in the post-pandemic period compared to children born before the pandemic. These findings highlight the important trade-offs inherent in pandemic infection control strategies and suggest that altered early-life microbial exposure can influence subsequent patterns of severe childhood infections. Whether this reflects reduced immunological priming, increased pathogen exposure during the rebound period, or a combination of both remains difficult to disentangle. Further pandemic preparedness should incorporate strengthened surveillance and preventive strategies for young children to anticipate and mitigate rebound waves of respiratory infections following periods of widespread transmission of pathogens.

The results emphasize the need for early treatment options and vaccines during epidemic periods, in order to adjust NPIs and minimize unintended consequences.

## Contributors

Conceptualization: HMD, HL, AS, FN, LD. Data analysis: HMD, HL. First draft of manuscript: HMD. Critical revision of manuscript: HL, AS, FN, LD, KK. All authors approved the final manuscript. HMD, HL, AS and FN directly accessed and verified the underlying data reported in the manuscript.

## Data sharing statement

The data used in this study are pseudonymized and sourced from Swedish healthcare registers and are not publicly available according to Swedish legislation. Interested researchers may seek ethical approval to access data from the appropriate Swedish public data holders according to the relevant legislation.

## Declaration of interests

FN owns AstraZeneca shares. HL is a member of executive committee in the Nordic PharmacoEpidemiological Network (NorPEN). AS, HMD, LD and KK declare no competing interests.
