# Supplementary material for: Post-pandemic respiratory infections in children born during or before the COVID-19 pandemic: a nationwide cohort study in Sweden
Source: eClinicalMedicine. 2026 Jul 28;98:104112. doi: 10.1016/j.eclinm.2026.104112 (PMC13446201; doi:10.1016/j.eclinm.2026.104112)
Supplement: Supplementary file 1 [file mmc1.docx]

**Post-pandemic respiratory infections in children born during or before the COVID-19 pandemic: a nationwide cohort study in Sweden**

Henrik Mellström Dahlgren, Huiqi Li, Ailiana Santosa, Leif Dotevall, Karsten Kötz, Fredrik Nyberg.

**Supplementary figures**

Supplementary figure 1. Study population of children born in Sweden between 1 March and 31 December in 2015, 2018 and 2020, with exclusions before start of follow-up at age 2 years.

Supplementary figure 2. Annual hospitalisation rate for pneumonia and influenza (ICD-10 codes J09-J18) per 100 000 inhabitants among children aged 0 – 4 years in Sweden, 2000-2024. Black line and points show observed annual hospitalization rates. Blue line shows the fitted segmented regression, with the estimated breakpoint (2020) indicated by the dashed vertical line. Data source: The National Board of Health and Welfare.

**Supplementary tables**

Supplementary Table 1. ICD-10 codes from primary or secondary diagnosis codes in the NPR used to define disease-specific hospitalisations from birth to baseline at age 2 and for outcomes between ages 2 and 4 years in children born in 2015, 2018 and 2020* in Sweden by birth cohort.

| **Outcome** | **Associated ICD-10 codes** |
| --- | --- |
| All-cause Lower Respiratory infection | A48.1;A70;B97.4;B97.5;B97.6;J09;J10;J10.0;J10.1;J10.2;J10.8;J10.9;J11;J11.0;J11.1;J11.2;J11.8;J12;J12.0;J12.1;J12.2;J12.3;J12.8;J12.9;J13;J14;J15;J15.0;J15.1;J15.2;J15.3;J15.4;J15.5;J15.6;J15.7;J15.8;J16;J16.0;J16.8;J20;J20.0;J20.1;J20.2;J20.3;J20.4;J20.5;J20.6;J20.7;J20.8;J20.9;J21;J21.0;J21.1;J21.8;J21.9;J91.0;P23.0;P23.1;P23.2;P23.3;P23.4 |
| All-cause Pneumonia | J10.0; J11.0; J12.0; J12.1; J12.2; J12.8; J12.9; J13; J14; J15.0; J15.1; J15.2; J15.3; J15.4; J15.5; J15.8; J15.9; J16.0; J16.8; J17; J18.0; J18.1; J18.8; J18.9; J22; J85.1 |
| Pneumonia bacterial | J13; J14; J15; J16.0; J85.1 |
| Pyothorax with or without fistula | J86.0; J86.9 |
| Influenza | J09; J10; J11 |
| Mycoplasma | A49.3; B96.0; J15.7; J20.5 |
| RSV | B97.4; J12.1; J20.5; J21.0 |
| Pertussis | A37.0; A37.1; A37.8; A37.9 |
| Invasive pneumococcal disease | A40.3; G00.1; J13; M00.1;  B95.3 in combination with one of: I30.1; K65.0; M00.8; O85; P23.6 |
| *Specifically born between 1 March and 31 December. | |

Supplementary table 2. Categorization of birth countries based on World Bank income classifications.

| **World bank classification** | **Categorization** | **Birth country** |
| --- | --- | --- |
| High-income | Sweden | Sweden |
| Low-income | Lower-middle-income (LMIC) | Afghanistan, Burkina Faso, Burundi, Central African Republic, Eritrea, Ethiopia, Gambia, Guinea, Guinea-Bissau, Democratic Republic of the Congo, North Korea, Liberia, Madagascar, Malawi, Mali, Mozambique, Niger, Rwanda, Sierra Leone, Somalia, Sudan, South Sudan, Syria, Chad, Togo, Uganda, Yemen |
| Lower-middle-income |  | Algeria, Angola, Arab Republic of Egypt, Bangladesh, Benin, Bhutan, Bolivia, Djibouti, Egypt, El Salvador, Côte d'Ivoire, Philippines, Morocco, Gaza Strip, Ghana, Haiti, Honduras, India, Indonesia, Iran, Cambodia, Cameroon, Cape Verde, Kenya, Kyrgyzstan, Congo, Laos, Lesotho, Mauritania, Mongolia, Myanmar, Nepal, Nicaragua, Nigeria, Pakistan, Palestine, Papua New Guinea, Solomon Islands, Samoa, São Tomé and Príncipe, Senegal, Sri Lanka, Eswatini, South Yemen, Tunisia, Ukraine, Uzbekistan, West Bank, Western Samoa, Vietnam, Zambia, Zimbabwe |
| Upper-middle-income | Middle-higher-income (MHIC) | Albania, Argentina, Armenia, Azerbaijan, Belarus, Bosnia and Herzegovina, Botswana, Brazil, Bulgaria, Colombia, Costa Rica, Dominica, Dominican Republic, Ecuador, Equatorial Guinea, Fiji, Gabon, Georgia, Grenada, Guatemala, Guyana, Iraq, Jamaica, Jordan, Yugoslavia, Kazakhstan, China, Kosovo, Cuba, Lebanon, Libya, North Macedonia, Malaysia, Maldives, Mauritius, Mexico, Moldova, Montenegro, Namibia, Panama, Paraguay, Peru, Romania, Russia, Saint Lucia, Saint Vincent and the Grenadines, Serbia, Serbia and Montenegro, Soviet Union, Suriname, South Africa, Tajikistan, Tanzania, Thailand, Tonga, Turkey, Turkmenistan, Venezuela, Belarus, Zanzibar |
| High-income |  | Antigua and Barbuda, United Arab Emirates, Australia, Bahamas, Bahrain, Barbados, Belgium, Brunei Darussalam, Chile, Cyprus, Denmark, Danzig, Estonia, Finland, France, Gibraltar, Greece, Hong Kong, Ireland, Iceland, Israel, Italy, Japan, British Virgin Islands, Canada, South Korea, Croatia, Kuwait, Latvia, Lithuania, Luxembourg, Malta, Monaco, Nauru, Netherlands, Norway, New Zealand, Oman, Palau, Poland, Portugal, Qatar, Saint Kitts and Nevis, San Marino, Saudi Arabia, Switzerland, Seychelles, Singapore, Slovakia, Slovenia, Spain, United Kingdom, Taiwan, Czech Republic, Czechoslovakia, Trinidad and Tobago, German Democratic Republic (East Germany), Germany, Hungary, Uruguay, United States, Austria |

Supplementary table 3. Distribution of birth, maternal and sociodemographic characteristics among excluded individuals. Q1, first quartile; Q3, third quartile 3; MHIC, middle-high income countries; LMIC, low-middle income countries.

|  |  | **Birth cohort** | | |
| --- | --- | --- | --- | --- |
| **Characteristic** | **Overall**  N = 30,910^1^ | **2015**  N = 14,544^1^ | **2018**  N = 9,558^1^ | **2020**  N = 6,808^1^ |
| **Sex** |  |  |  |  |
| Male | 15,846 (51%) | 7,486 (51%) | 4,919 (51%) | 3,441 (51%) |
| Female | 15,064 (49%) | 7,058 (49%) | 4,639 (49%) | 3,367 (49%) |
| **Birth weight in grams** | 3,410 (3,022, 3,780) | 3,330 (2,980, 3,725) | 3,440 (3,130, 3,815) | 3,433 (3,075, 3,862) |
| Missing | 30,447 | 14,382 | 9,415 | 6,650 |
| **Gestation age in days** | 278 (269, 285) | 279 (271, 285) | 276 (267, 285) | 277 (266, 283) |
| Missing | 29,601 | 13,783 | 9,262 | 6,556 |
| **Gestation age (weeks + days)** |  |  |  |  |
| Normal (37+0–41+6) | 1,080 (3.5%) | 649 (4.5%) | 224 (2.3%) | 207 (3.0%) |
| Extremely preterm (22+0–27+6) | 33 (0.1%) | 16 (0.1%) | 9 (<0.1%) | 8 (0.1%) |
| Very preterm (28+0–31+6) | 31 (0.1%) | 15 (0.1%) | 9 (<0.1%) | 7 (0.1%) |
| Moderately preterm (32+0–36+6) | 125 (0.4%) | 63 (0.4%) | 37 (0.4%) | 25 (0.4%) |
| Post-term (≥42+0) | 39 (0.1%) | 17 (0.1%) | 17 (0.2%) | 5 (<0.1%) |
| Missing | 29,602 (96%) | 13,784 (95%) | 9,262 (97%) | 6,556 (96%) |
| **Residential area type** |  |  |  |  |
| 5· Very good socioeconomic conditions | 426 (1.4%) | 219 (1.5%) | 132 (1.4%) | 75 (1.1%) |
| 4· Good socioeconomic conditions | 2,277 (7.4%) | 1,000 (6.9%) | 761 (8.0%) | 516 (7.6%) |
| 3· Mixed areas | 1,371 (4.4%) | 581 (4.0%) | 476 (5.0%) | 314 (4.6%) |
| 2· Socioeconomic challenges | 673 (2.2%) | 308 (2.1%) | 205 (2.1%) | 160 (2.4%) |
| 1· Major socioeconomic challenges | 703 (2.3%) | 315 (2.2%) | 226 (2.4%) | 162 (2.4%) |
| Missing | 25,460 (82%) | 12,121 (83%) | 7,758 (81%) | 5,581 (82%) |
| **Household size (numbers of persons)** |  |  |  |  |
| **≤**3 | 2,229 (7.2%) | 922 (6.3%) | 708 (7.4%) | 599 (8.8%) |
| 4-7 | 2,790 (9.0%) | 1,308 (9.0%) | 934 (9.8%) | 548 (8.0%) |
| ≥8 | 454 (1.5%) | 216 (1.5%) | 158 (1.7%) | 80 (1.2%) |
| Missing | 25,437 (82%) | 12,098 (83%) | 7,758 (81%) | 5,581 (82%) |
| **Maternal birth country** |  |  |  |  |
| Born in Sweden | 3,349 (11%) | 1,589 (11%) | 1,035 (11%) | 725 (11%) |
| Born in MHIC | 11,347 (37%) | 5,229 (36%) | 3,616 (38%) | 2,502 (37%) |
| Born in LMIC | 14,599 (47%) | 7,133 (49%) | 4,407 (46%) | 3,059 (45%) |
| Unknown | 1,615 (5.2%) | 593 (4.1%) | 500 (5.2%) | 522 (7.7%) |
| **Maternal age, years Median (Q1, Q3)** | 29.0 (25.0, 33.0) | 29.0 (25.0, 33.0) | 30.0 (26.0, 34.0) | 30.0 (26.0, 34.0) |
| Missing | 1,608 | 594 | 498 | 516 |
| **Maternal smoking** |  |  |  |  |
| Nonsmoker | 587 (1.9%) | 239 (1.6%) | 179 (1.9%) | 169 (2.5%) |
| Smoking according to pregnancy | 30,305 (98%) | 14,301 (98%) | 9,373 (98%) | 6,631 (97%) |
| Missing | 18 (<0.1%) | 4 (<0.1%) | 6 (<0.1%) | 8 (0.1%) |
| **Maternal education** |  |  |  |  |
| Tertiary | 2,250 (7.3%) | 1,032 (7.1%) | 704 (7.4%) | 514 (7.5%) |
| Secondary | 1,197 (3.9%) | 578 (4.0%) | 365 (3.8%) | 254 (3.7%) |
| Primary/Missing | 27,463 (89%) | 12,934 (89%) | 8,489 (89%) | 6,040 (89%) |
| ^1^n (%); Median (Q1, Q3) | | | | |

Supplementary table 3· Baseline outcomes among children excluded due to missing covariate data, by birth cohort. RSV, respiratory syncytial virus.

|  | **Birth cohort** | | |  |
| --- | --- | --- | --- | --- |
|  | **2015**  N = 14,281*^1^* | **2018**  N = 9,298*^1^* | **2020**  N = 6,602*^1^* | **p-value***^2^* |
| **Hospitalisations due to respiratory infections** |  |  |  |  |
| All-cause Lower respiratory tract infection | 95 (0.7%) | 41 (0.4%) | 26 (0.4%) | 0.014 |
| All-cause Pneumonia | 32 (0.2%) | 17 (0.2%) | 15 (0.2%) | 0.8 |
| Pneumonia - bacterial | 6 (<0.1%) | 7 (<0.1%) | 3 (<0.1%) | 0.6 |
| Invasive pneumococcal infection | 1 (<0.1%) | 0 | 0 | >0.9 |
| Pyothorax | 0 | 0 | 0 |  |
| Influenza | 8 (<0.1%) | 5 (<0.1%) | 1 (<0.1%) | 0.5 |
| Mycoplasma | 4 (<0.1%) | 3 (<0.1%) | 0 | 0.5 |
| Pertussis | 1 (<0.1%) | 0 | 0 | >0.9 |
| RSV | 31 (0.2%) | 16 (0.2%) | 11 (0.2%) | 0.6 |
| *^1^*n (%) *^2^*Pearson's Chi-squared test | | | |  |

Supplementary table 4· Cumulative incidence of infection-related hospitalisations and all-cause death among children excluded due to missing covariate information between ages 2 and 4 years across birth cohorts*. RSV, respiratory syncytial virus.

|  | **Birth cohort** | | |  |
| --- | --- | --- | --- | --- |
| **Respiratory outcome** | **2015**  N = 14,281*^1^* | **2018**  N = 9,298*^1^* | **2020**  N = 6,602*^1^* | **p-value***^2^* |
| All-cause Lower respiratory tract infection | 42 (0·3%) | 28 (0·3%) | 55 (1·8%) | <0·001 |
| All-cause Pneumonia | 29 (0·2%) | 19 (0·2%) | 29 (0·4%) | 0·004 |
| Pneumonia - bacterial | 13 (<0·1%) | 5 (<0·1%) | 11 (0·2%) | 0·075 |
| Invasive pneumococcal infection | 1 (<0·1%) | 2 (<0·1%) | 2 (<0·1%) | 0·4 |
| Pyothorax | 0 | 2 (<0·1%) | 0 | 0.14 |
| Mycoplasma | 3 (<0·1%) | 2 (<0·1%) | 4 (<0·1%) | 0.3 |
| Influenza | 10 (<0·1%) | 0 | 13 (0·2%) | <0·001 |
| RSV | 7 (<0·1%) | 12 (0·1%) | 23 (0·3%) | <0·001 |
| Pertussis | 0 | 0 | 0 |  |
| All-cause death | 1 (<0·1%) | 1 (<0·1%) | 1 (<0·1%) | 0·8 |
| *^1^*n (%) | | | | |
| *^2^*Pearson's Chi-squared test *Specifically born between March and December | | | | |

Supplementary Table 5. Number and proportion of infection-related hospitalisation and all-cause death between ages 2 and 4 years in children born in 2015, 2018 and 2020* in Sweden by birth cohort among a subset of small households (≤3 persons in household). RSV, respiratory syncytial virus.

|  | **Birth cohort** | | |  |
| --- | --- | --- | --- | --- |
| **Outcome** | **2015** N = 35,908*^1^* | **2018** N = 36,901*^1^* | **2020** N = 36,413*^1^* | **p-value***^2^* |
| All-cause Lower respiratory tract infection | 220 (0·6%) | 219 (0·6%) | 520 (1·4%) | <0·001 |
| All-cause Pneumonia | 155 (0·4%) | 139 (0·4%) | 352 (1·0%) | <0·001 |
| Pneumonia - bacterial | 52 (0·1%) | 45 (0·1%) | 145 (0·4%) | <0·001 |
| Invasive pneumococcal infection | 6 (<0·1%) | 7 (<0·1%) | 17 (<0·1%) | 0·025 |
| Pyothorax | 2 (<0·1%) | 1 (<0·1%) | 11 (<0·1%) |  |
| Mycoplasma | 11 (<0·1%) | 20 (<0·1%) | 36 (<0·1%) | 0·001 |
| Influenza | 44 (0·1%) | 11 (<0·1%) | 118 (0·3%) | <0·001 |
| RSV | 62 (0·2%) | 102 (0·3%) | 219 (0·6%) | <0·001 |
| Pertussis | 0 (0%) | 0 (0%) | 1 (<0·1%) |  |
| All-cause death | 5 (<0·1%) | 5 (<0·1%) | 6 (<0·1%) | >0·9 |
| *^1^*n (%) | | | | |
| *^2^*Pearson's Chi-squared test *Specifically born between March and December. | | | | |
